# Rapid Concentration of Ga-68 and Proof-of-Concept Microscale Labeling of [^68^Ga]Ga-PSMA-11 in a Droplet Reactor

**DOI:** 10.3390/molecules29194572

**Published:** 2024-09-26

**Authors:** Yingqing Lu, Philip H. Chao, Jeffrey Collins, R. Michael van Dam

**Affiliations:** 1 Crump Institute for Molecular Imaging, University of California Los Angeles (UCLA), Los Angeles, CA 90095, USA; 2 Department of Molecular & Medical Pharmacology, University of California Los Angeles (UCLA), Los Angeles, CA 90095, USA

**Keywords:** radiometal, radionuclide concentration, Gallium-68 (Ga-68), [^68^Ga]Ga-PSMA-11, microscale radiosynthesis, droplet reactor

## Abstract

The radiometal gallium-68 (Ga-68) has garnered significant interest due to its convenient production via compact and widely available generators and the high performance of ^68^Ga-labeled compounds for positron-emission tomography (PET) imaging for cancer diagnosis and management of patients undergoing targeted radionuclide therapy. Given the short half life of Ga-68 (68 min), microfluidic-based radiosynthesis is a promising avenue to establish very rapid, efficient, and routine radiolabeling with Ga-68; however, the typical elution volume of Ga-68 from a generator (4–10 mL) is incompatible with the microliter reaction volumes of microfluidic devices. To bridge this gap, we developed a microscale cartridge-based approach to concentrate Ga-68. By optimizing cartridge design, resin type, resin mass, and eluent composition, Ga-68 was reliably concentrated from ~6 mL to ~80 µL with high recovery efficiency (>97%, n = 14). Furthermore, this method is suitable for both single- and dual-generator setups. To demonstrate suitability of the concentrated radiometal for radiolabeling, we performed microdroplet synthesis of [^68^Ga]Ga-PSMA-11, achieving high radiochemical yield (83 ± 11%, n = 3), excellent radiochemical purity (>99%), and high apparent specific activity (255–320 MBq/μg). The entire process, including Ga-68 concentration, radiosynthesis, purification, and formulation, was completed in 12 min. Starting with activity of 0.81–0.84 GBq, 0.51–0.64 GBq of product was produced, sufficient for multiple patient doses. This work paves the way to clinical-scale production of other ^68^Ga-labeled compounds using droplet microreactor methods, or high-throughput labeling optimization or compound screening of ^68^Ga-labeled probes using droplet reaction arrays.

## 1. Introduction

Due to their convenient production and theranostic potential, positron-emitting radiometals produced via compact generators have garnered significant interest in recent years [1]. Notable radionuclides generated in this manner include gallium-68 (Ga-68), rubidium-82, and copper-62 [2]. Among these, Ga-68 stands out due to its high positron yield (89%) and moderate half-life (68 min) that affords sufficient time for radiolabeling and patient imaging. Numerous ^68^Ga-labeled imaging agents, including those based on small molecules, peptides, antibodies, and particles, have been developed for the visualization of various cancers and physiological conditions [3,4].

Radiometals such as Ga-68 are also increasingly recognized for their theranostic potential, where the same or similar targeting molecules can be labeled with Ga-68 for imaging or with a therapeutic radionuclide (e.g., Y-90, Lu-177, which have similar coordination chemistry to Ga-68) for treatment. The ^68^Ga-labeled imaging compound can play a role in patient care, aiding in disease staging, calculating patient-specific dosimetry for optimized therapeutic dosing, or monitoring the patient’s response to treatment [5], or can play a role in therapeutic development by providing information about biodistribution and dosimetry. The therapeutic counterpart is designed to accumulate at disease sites, and decay of the radionuclide emits short-range beta particles, alpha particles, or Auger electrons that induce DNA damage and lead to localized cell death [6].

This theranostic approach is exemplified by FDA-approved imaging agents targeting somatostatin receptors, such as [^68^Ga]Ga-DOTA-TATE (Netspot™), and therapeutic agents such as [^177^Lu]Lu-DOTA-TATE (LUTATHERA^®^) for treating patients with neuroendocrine tumors [7]. Another example is the approval of the radiotherapeutic [^177^Lu]Lu-PSMA-617 (Pluvicto™) in 2022 for prostate cancer (targeting prostate specific membrane antigen, PSMA), following the approval of its imaging probe [^68^Ga]Ga-PSMA-11 (Locametz^®^) in 2021 [8,9]. 

Recent advancements in microfluidics have opened new avenues for improving the efficiency and cost-effectiveness of radiosynthesis by operating in microliter volumes [10,11,12,13,14,15]. Microscale devices enhance reaction performance through more efficient heat transfer and rapid reagent mixing. Microfluidic reactors, with typical volumes on the order of 10s of microliters, significantly reduce reagent consumption, up to 2–3 orders of magnitude compared to conventional methods [16,17,18], thus lowering production costs and simplifying downstream purification. Additionally, synthesizing radiotracers in such small volumes can increase molar or specific activity due to minimal non-radioactive isotope contamination from solvent, reagents, and/or vials, or can improve apparent molar or specific activity by reducing precursor amounts in processes where leftover precursors (or chemically similar side-products) cannot be removed [19,20,21,22,23,24,25], leading to improved image quality in PET scans [23] and a reduced chance of pharmacologic effects [26].

Despite these advantages, the majority of microfluidic radiochemistry research has concentrated on ^18^F- and ^11^C-labeled agents, with many fewer studies exploring microscale radiometal labeling, particularly using the short half-life isotope Ga-68. One significant challenge is the lack of a rapid and efficient method for concentrating radiometals, which is crucial for bridging the gap between the initial mL-scale volume of the radiometal generator source and the µL-scale radiosynthesis on microfluidic devices. Recently, Zhang et al. developed a custom polydimethylsiloxane (PDMS)-based microfluidic chip (RAPID chip) capable of concentrating 4 mL of Ga-68 from a generator down to 200 µL using on-chip 8 mg of strong cation exchange (SCX) and 10 mg of strong anion exchange (SAX) resins, which was combined with 200 µL of a precursor solution in a total reaction volume of 400 µL in a flow-based radiosynthesis of [^68^Ga]Ga-PSMA-11 [24]. During the concentration process, the large volume of Ga-68 from the generator is flowed through, trapping the [^68^Ga]Ga^3+^ on the SCX resin, which is subsequently eluted with a smaller volume of eluent solution. Although the concentration process could be accomplished in ~12 min, the overall activity recovery of the concentration process was moderate (73.7 ± 3.8%, n = 5), representing > 25% activity loss prior to the radiolabeling step. More recently, Mallapura et al. employed a commercial microfluidic cassette-based radiosynthesizer (iMiDEV, PMB Alcen, Peynier, France) to achieve an improved overall activity recovery of 89.6% for Ga-68 concentration (trapping efficiency: >98.5%; elution efficiency: 91 ± 6%, n = 4) using SCX resin and 200 µL of eluent. This was accomplished by incorporating reverse trapping and pulse flow techniques, followed by mixing with 170 µL of precursor solution in a total volume of ~370 µL for radiolabeling [27]. However, further concentrating Ga-68 to 10s of microliters for use in microfluidic devices with smaller reaction volumes, such as droplet reactors [16,17,20], remains unexplored.

**Figure 1:**
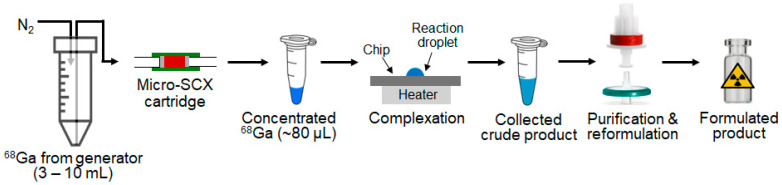
Process flow for the concentration of Ga-68 using a micro-SCX cartridge and the subsequent ^68^Ga-tracer production via microdroplet radiochemistry.

To address this limitation, we optimized various parameters, including resin types, resin mass, cartridge geometry, flow rate, and the type and volume of eluent solutions, to develop a microscale process to achieve rapid and efficient Ga-68 concentration with a final volume of 10s of microliters. After establishing the optimal concentration protocol, we further evaluated the capabilities of this miniaturized cartridge to concentrate an entire batch of Ga-68 (i.e., full generator elution). Since the output of this research generator available to us was somewhat low, we also explored the ability to combine the output of multiple generators. To confirm reactivity of the resulting concentrated Ga-68, we integrated it into a synthesis workflow (Figure 1), transferring it to a downstream microdroplet-based radiosynthesizer, and showed that rapid and efficient radiosynthesis of [^68^Ga]Ga-PSMA-11 was possible.

## 2. Results

### 2.1. Impact of Cartridge Design on Flow Rate

Due to the short half-life of Ga-68, the speed of the concentration process can have a significant impact on the overall performance. Cartridges were fabricated by packing resin into tubing, and flow rate through the cartridge was compared for different resin types, resin mass, and tubing inner diameter (ID) (Table S1). Aiming for an upper limit of 15 min for the entire concentration process (trapping, washing, eluting) to minimize activity loss due to Ga-68 decay over time, and assuming starting volumes of Ga-68 up to 10 mL (plus ~1 mL volume or less for washing and elution), we selected ~750 µL/min as a lower limit of acceptable flow rate. All cartridges, with the exception of 5 mg Oasis MCX resin in 0.03’’ ID tubing, met this criteria. As expected, the flow rate decreased as resin mass was increased or tubing diameter decreased (leading to lengthening of the resin bed compared to larger diameter tubing). We also observed that flow rates were higher for the Chromafix PS-H^+^ resin versus the Oasis MCX resin, likely due to the larger particle size (100 µm versus 60 µm), which leads to lower flow resistance.

### 2.2. Preliminary Evaluation of Resin Mass

A series of preliminary experiments was performed to help identify which cartridge characteristics (e.g., resin mass, tubing inner diameter (ID)) should be prioritized in order to achieve high trapping and elution efficiency (data summarized in Table S2). 1 mL aliquots of the Ga-68 source were used for tapping, and subsequent elution was performed with a single 200 µL volume of 0.9 N HCl in 90% (*v/v*) EtOH/H_2_O (conditions adopted from ref. [28]). With only 3 mg of resin, similar trapping efficiencies of 83% and 81% were found on 0.03″ and 0.04″ ID tubing, respectively, but the elution efficiency was higher for the narrower cartridge design (i.e., 94% for 0.03″ ID tubing versus 71% for 0.04″ ID tubing). In general, increasing the resin mass increased the trapping efficiency and led to lower elution efficiency. However, packing larger resin masses (i.e., ≥7–9 mg) in smaller ID tubing (i.e., 0.03″) is impractical due to reduced flow rates, so larger masses had to be packed in larger tubing (0.04″ or 0.06″ ID), resulting in high trapping efficiency, but elution efficiency decreased. For further studies, we selected cartridge geometries with high trapping efficiency (i.e., cartridges packed with ≥7 mg resin) and optimized the elution protocol.

### 2.3. Concentration with Narrow Cartridges (0.04″ ID)

In cartridges packed with 0.04″ ID tubing, starting activities ranged from 20–40 MBq in 1 mL of 0.1 N HCl. (Scale-up of activity amount and volume was conducted in later experiments to confirm the trapping capacity of the cartridges.) During trapping, the Ga-68 was driven by N_2_ gas at 20 psi. Elution was performed with different eluent solutions driven at 5 psi and different numbers of elution steps (i.e., 10 µL volume increments) (Table S3).

Trapping efficiency was nearly quantitative across all resin types and masses, with only slightly lower performance of 7 mg cartridges compared to 9 mg. The cumulative elution efficiency in all cases was significantly higher with Chromafix PS-H^+^ resin than with Oasis MCX resin. For this reason, we focused on the Chromafix PS-H+ resin for the remainder of our work. The acetone-based eluent yielded an elution efficiency of 96 ± 0% (n = 2) for the 7 mg cartridge and 96 ± 1% (n = 2) for the 9 mg cartridge after six elution steps. However, when fewer elution portions were used, the elution efficiency dropped significantly: 68–74% for four elution steps or 5–12% for two elution steps. Using 0.13 N HCl in 5 M NaCl as the eluent, the elution efficiency was lower, reaching a maximum of 87 ± 10% (n = 2) with the 9 mg cartridge and 6 elution steps. For the 0.9 N HCl in 90% (*v/v*) EtOH/H_2_O eluent, the efficiency was very low, reaching at most only 10 ± 9% (n = 2) for the 9 mg cartridge and 6 elution steps.

We then wanted to confirm whether the optimized condition would be effective in concentrating the full amount of Ga-68 solution from the generator (i.e., containing up to 970 MBq for our generators). First, we verified that larger starting volumes do not negatively impact trapping and elution behavior. Ga-68 stock solutions (37–74 MBq) were diluted from 1 mL to 10 mL with 0.1 N HCl, and trapping and elution experiments were repeated (results shown in Table S4). Trapping and elution efficiency remained high (i.e., 99 ± 0%, n = 2, and 96 ± 0%, n = 2, respectively).

Next, we tested higher starting activity in a 10 mL volume (Table S4). For a starting activity of 440 MBq, 6 mL of generator eluent was diluted to 10 mL with 0.1 N HCl, achieving 99% (n = 1) trapping efficiency and 93% (n = 1) elution efficiency. We then performed trapping and elution experiments with even higher starting activities (760–970 MBq) in 6 mL volumes directly from the generator but without dilution in order to time the entire concentration process in a realistic way. Trapping efficiency remained quantitative (100 ± 0%, n = 2), and elution efficiency remained high (93 ± 0%, n = 2). Overall, the concentration of 6 mL of Ga-68 to 60 μL took a total of ~ 9 min (i.e., ~ 4 min for the trapping process and 5 min for the 6 elution operations combined). These results confirmed the ability to efficiently concentrate the entire batch of Ga-68 from the generator.

### 2.4. Concentration with Wider Cartridges (0.06″ ID)

Next, to explore the possibility of reducing the processing time, we performed studies with cartridges packed into larger-diameter tubing, which we had previously observed to have significantly increased flow rates. We prepared cartridges using 5 to 12 mg of resin packed into 0.06″ ID tubing. Trapping experiments were performed starting with 6 mL of Ga-68 in 0.1 N HCl (52–81 MBq, obtained by dilution of activity from the generator) flowed through the cartridge at 12 psi. As summarized in Table 1, trapping efficiency improved significantly as the resin mass increased, ranging from 76 ± 5% (n = 2) for 5 mg of resin to 94 ± 1% (n = 2) for 12 mg of resin. We attempted experiments at lower pressures (and flow rates) and found a slight improvement was possible. At 8 psi driving pressure, trapping efficiency was 96 ± 2% (n = 2) and took only ~2 min.

**Figure 2:**
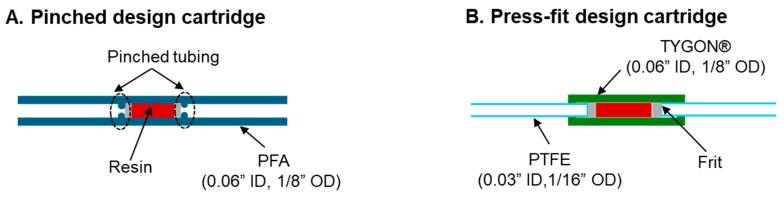
Schematics of the different cartridge designs. (**A**) Pinched design cartridge. (**B**) Press-fit design cartridge.

We next considered the repeatability and practicality of the concentration process. Some fabricated cartridges had significantly different than average flow performance, perhaps due to the difficulty of repeatably pinching the tubing to secure the frits and resin. To address this issue, we constructed cartridges using an alternate approach to secure the frits and resin, namely the press-fit design shown in Figure 2. Trapping efficiency was even higher for these cartridges (100 ± 0%, n = 4) when operated at a slightly lower driving pressure of 6 psi (which allows for greater interaction time between Ga-68 and the trapping resin), with a trapping time of ~3.5 min (Table 1). Next, the elution process using the acetone-based eluent was optimized. To simplify and accelerate the elution process, we increased the volume of each elution step but reduced the number of steps. Instead of performing 6 × 10 µL elution steps, we eluted Ga-68 using a single 60 µL volume followed by a second elution step, where the volume was varied (20–60 µL) to determine the minimum amount needed (Table 2). With just one 60 µL elution (no second elution), we achieved a moderate elution efficiency of 80–84% (n = 17, averaged over all single-generator tests). When followed by a second elution, the efficiency increased, with higher performance for higher volumes, reaching 98 ± 2% (n = 11) with 60 µL as the volume of the second elution.

The modified elution strategy significantly shortened the process. The total elution time was ~1 min, with the first 60 µL elution taking ~40 s and the second elution taking ~20 s. Overall, the modified concentration process was completed in ~4.5 min (~3.5 min for trapping and ~1 min for elution), shrinking the Ga-68 volume from the initial 6 mL to ~80 µL. Note that the actual recovered volume is lower than the expected 120 µL; we assume that 40 µL remains within the cartridge and/or evaporates during the process. 

Since the output of this research generator accessible to us was fairly low, we explored the possibility of scaling up reaction by combining the output of multiple generators. By collecting fractions, we discovered that > 93% of the activity from a generator is recovered in the middle fraction (i.e., we collected 3.2 mL of the generator output after discarding the first 1.2 mL). We combined the fractioned Ga-68 from two generators (aged 42 weeks and 66 weeks at the time of the experiment), giving a total of ~6.4 mL of 0.1 N HCl-based solution containing 932–973 MBq total. The combined activity was trapped on cartridges with quantitative efficiency (100 ± 0%, n = 3), followed by a high elution efficiency (97 ± 3%, n = 3) within a similar time of ~4.5 min.

### 2.5. Proof-of-Concept [^68^Ga]Ga-PSMA-11 Synthesis in a Droplet Reactor

With the concentrated microvolume Ga-68 in hand, we proceeded with a proof-of-concept labeling experiment using the widely employed clinical radiopharmaceutical [^68^Ga]Ga-PSMA-11. The aim was to verify the ability to translate macroscale ^68^Ga-labeling protocols onto our microdroplet reactor and to assess the quality of the concentrated Ga-68. 

After the concentration process (from dual Ga-68 generators), we obtained ~80 μL of activity in 0.05 N HCl in a 98% (*v/v*) acetone/H_2_O solution, collected in a 2 mL microcentrifuge tube. Further volume reduction was performed by transferring the solution to a microdroplet reactor chip, where a subsequent ^68^Ga-labeling reaction can be performed.

The chip consists of a 25 mm square piece of Teflon-coated silicon chip, patterned with reaction sites comprising 4 mm circular regions where the Teflon has been removed to expose the hydrophilic silicon. The chip was operated on a temperature-controlled heating platform, as previously described [20]. Each radiosynthesis utilized a single reaction site for the entire synthesis process, including acetone evaporation from the concentrated Ga-68 solution and the subsequent ^68^Ga radiolabeling. Each reaction site was used only once, and once all reaction sites had been used, the chip was replaced with a new one. 

To prevent over-heating of dried Ga-68, 10 μL of saline was first added to the chip, and then the concentrated Ga-68 solution was manually pipetted from the microcentrifuge tube to the chip in 20 μL increments, each dried at 60 °C before adding the next, until the entire batch was processed. During this process, 88 ± 13% (n = 3) of Ga-68 was successfully transferred (i.e., ~12% loss in microcentrifuge tube and pipette tip). The evaporation took ~2 min, leaving around 5 μL of the saline/GGa-68 mixture at the reaction site. (The volume was estimated based on a mock evaporation study via a micro-pipettor, and details can be found in Supplemental Information). Following Zhang et al.’s optimized complexation condition for preparing [^68^Ga]Ga-PSMA-11 on a microfluidic system [24], but in a reaction volume ~27 times smaller, we added 10 μL of a stock solution containing 2 μg of precursor to the reaction site and heated the resulting mixture at 95 °C for 1 min. A summary of the radiosynthesis performance is included in Table 3. Analysis of the crude reaction mixture via radio-TLC showed a quantitative complexation efficiency (100 ± 0%, n = 3). Due to the small reaction volume, we were not able to measure the pH in situ, but based on a mock radiolabeling reaction with all constituents except the Ga-68 (details in Supplemental Information), we confirmed that the pH of the reaction solution is 4. Following cartridge purification and formulation, the final product was obtained with a high radiochemical yield (RCY) of 83 ± 11% (n = 3) and radiochemical purity > 99%. The overall activity yield was 73 ± 10% (n = 3), resulting in a final product of 0.51–0.64 GBq [13.8–17.5 mCi]. The stability of the formulated product at room temperature was assessed by radio-HPLC over a duration of 2 h, and no significant changes were observed, indicating good stability and absence of radiolysis. At the end of synthesis (EOS), a high apparent specific activity was achieved (255–320 MBq/μg). The total preparation time was ~12 min, including ~4.5 min for Ga-68 concentration, ~2 min for acetone evaporation, ~2 min for radiolabeling and product collection from the chip reactor, and ~3.5 min for C18 cartridge-based purification and formulation. Details of the entire radiosynthesis process are summarized in Table S5.

## 3. Discussion

In this work, we successfully developed two micro-cartridge-based methods to concentrate generator-produced Ga-68 from volumes up to 10 mL down to 60–80 µL. Using miniaturized, custom-fabricated SCX cartridges, we optimized the process, evaluating various cartridge designs, resin types, resin masses, trapping conditions, and elution conditions. The optimal setup enabled nearly quantitative trapping and elution of Ga-68.

Reduction in output volume (>3× lower) compared to other approaches for Ga-68 concentration reported for microfluidic platforms (Table 4) [24,27]. In a modified approach, the concentration time was further reduced to ~4.5 min using a press-fit design micro-cartridge (packed with 12 mg of Chromafix PS-H^+^ resin in 0.06″ ID tubing) and the same acetone-HCl eluent. Furthermore, the overall activity recovery efficiency was improved (98 ± 2%, n = 11), achieving the highest activity recovery performance among reported Ga-68 concentration methods for microfluidic devices [24,27,32]. The final concentrated volume was slightly larger with the second approach (~80 µL), but still a significant reduction over other reported methods.

The latter method was furthermore shown to be compatible with combined output from dual Ga-68 generators, exhibiting nearly identical performance as a single generator. This could be useful in facilities that are trying to stretch the usage of the Ga-68 generators after they no longer produce enough activity for clinical doses, but when combined may provide sufficient activity. 

In our initial studies, we showed concentration of Ga-68 (up to 970 MBq from a single generator) from 6 mL down to ~60 µL in ~9 min, with an overall recovery efficiency of 93 ± 0% (n = 2), using a pinched design SCX micro-cartridge (packed with 9 mg of Chromafix PS-H^+^ resin in 0.04″ ID tubing) and an acetone-HCl eluent. This represents a significant

Moreover, the use of an acetone-based eluent allows for rapid further concentration of Ga-68 via evaporation at moderate temperature on a droplet reactor chip to achieve a final activity volume of ~5 µL. In principle, instead of adding saline to the chip prior to the addition of concentrated Ga-68, other buffer solutions could be added depending on the needs of the downstream labeling reaction. This adaptability is a considerable advantage over methods that use DI water or high-concentration NaCl solutions as eluents [24,27], which can be difficult and time-consuming to remove or adapt to different solvent conditions. One potential concern with using an acetone-based eluent is residue in the final product, but this was simply mitigated by choosing a heating temperature of 60 °C (above acetone’s boiling point of 56 °C). This process removed most of the acetone, leaving only ~5 µL of saline on the chip after evaporation. Even if this residual volume contained 100% acetone (which is extremely unlikely given the much higher boiling point of saline of ~100 °C compared to acetone and the lack of azeotrope formation between acetone and water [33]), the calculated maximum acetone concentration in the final formulated product (16.5 mL) would be 0.03% (*v/v*, 300 ppm), well below the permitted 5000 ppm for residual acetone in radiopharmaceutical formulations [34].

In proof-of-concept radiosynthesis using the concentrated Ga-68, [^68^Ga]Ga-PSMA-11 was successfully prepared rapidly and with high efficiency in a microdroplet reactor. Starting from Ga-68 from dual generators, the final product was achieved with an RCY of 83 ± 11% (n = 3) within a total preparation time of only 12 ± 0 min (n = 3). This represents the best performance among reported methods that involve a Ga-68 purification/concentration process. Compared to reported methods using unprocessed Ga-68, the performance was a little bit lower due to the loss of ~12% of the activity when transferring the concentrated Ga-68 onto the droplet reactor chip. This loss is likely to be reduced in future automated setups, where the eluted concentrated Ga-68 will be directly dispensed onto the microdroplet reaction chip, eliminating the need for intermediate storage in a transfer vial.

The final amount of product was 0.51–0.64 GBq [13.8–17.5 mCi], sufficient for multiple patient doses (3–7 mCi per patient [35]). Radiochemical purity was excellent (100 ± 0%, n = 3) and apparent specific activity was high (255–320 MBq/μg). In the microdroplet format, despite using 5–13× less precursor than other methods, a very high precursor concentration of 132 μM was achieved, 7–50× higher than all other microfluidic-based approaches and 27–67× higher than reported macroscale radiosyntheses. Since we have not yet optimized the precursor amount, reductions may be possible while still achieving good reaction performance, which would significantly increase the apparent specific activity.

The Ga-68 concentration method using micro-cartridges developed in this study can be further applied to other ^68^Ga-based radiopharmaceuticals, such as [^68^Ga]Ga-DOTA-TATE, [^68^Ga]Ga-DOTA-NOC, [^68^Ga]Ga-DOTA-TOC [36] and numerous other compounds in clinical trials globally [37]. Furthermore, this approach could also be adapted for concentration of cyclotron-produced Ga-68, which is of high interest due to the ability to produce larger amounts of the radionuclide and thus more batches of the labeled tracer per production run.

## 4. Materials and Methods

### 4.1. Materials

Anhydrous methanol (MeOH, 99.8%), ethanol (EtOH, 99.5%), and ammonium acetate (≥99.99% trace metal basis) were purchased from Sigma Aldrich (St. Louis, MO, USA). Acetone (GC ≥ 99.9%), hydrochloric acid (HCl, 37 wt.% in H_2_O, 99.999% trace metal basis), and 4-(2-hydroxyethyl)piperazine-1-ethanesulfonic acid (HEPES, catalog #PHG0001) were purchased from Honeywell Research Chemicals (Morris Plains, NJ, USA). PSMA-11 precursor (catalog #9920) and reference standard for [⁶⁸Ga]Ga-PSMA-11 (catalog #9922) were purchased from ABX Advanced Biochemical Compounds GmbH (Radeberg, Germany). DI water was obtained from a Milli-Q water purification system (EMD Millipore Corporation, Berlin, Germany). Phosphate buffered saline (PBS, catalog #BP243820) and pH test paper (pH range of 0–13, catalog #205522) were purchased from Fisher Scientific (Waltham, MA, USA). Ga-68 was obtained in 3.2–6.4 mL of 0.1 N HCl from single or dual generator(s) (IGG100, catalog #3131-0900, 50 mCi rating, Eckert & Ziegler, Valencia, CA, USA) available for research use at the UCLA Ahmanson Biomedical Cyclotron Facility. Centrifuge tubes with screw caps (5 and 25 mL, BioBased) and microcentrifuge tubes (0.5 and 2 mL, Safe-Lock) were purchased from Eppendorf (Hamburg, Germany). Chromafix PS-H^+^ (220 mg, 100 µm particle size, catalog #731861) was obtained from Macherey-Nagel (Bethlehem, PA, USA). Oasis MCX Plus Short (225 mg, 60 µm particle size, catalog #186003516) was purchased from Waters Corporation (Milford, MA, USA). C18 Light cartridges (catalog #WAT023501) were purchased from Waters Corporation (Milford, MA, USA).

The PSMA-11 precursor stock solution was prepared by dissolving 1 mg of PSMA-11 precursor with 1 mL of DI water to achieve a concentration of 1 µg/µL and was stored in the freezer. For further radiosynthesis experiments, this stock solution was diluted to the required concentration using 1.5 M HEPES buffer (obtained from the UCLA Ahmanson Biomedical Cyclotron Facility). 1 mg of PSMA-11 reference standard was dissolved in 1 mL of 0.1% TFA in deionized water (*v/v*) and was also stored in the freezer for future analysis.

### 4.2. Cartridge Fabrication

Cartridges were fabricated by packing resin between frits inside tubing of varying inner diameters (ID) and outer diameters (OD). Short segments of 1/16″ OD ethylene tetrafluoroethylene (ETFE) tubing (IDEX Health and Science, Wallingford, CT, USA) with 0.03″ ID (catalog #1528L, IDEX) or 0.04″ ID (catalog #1517L, IDEX), or polytetrafluoroethylene (PTFE) tubing with 1/16″ OD 0.03″ ID (catalog #QL694, Cole Parmer, Vernon Hills, IL, USA), were used. Additionally, larger perfluoroalkoxy (PFA) tubing (catalog #AP–231SH, Zeus Industrial Products, Inc., Orangeburg, SC, USA) and high-chemical-resistance Tygon^®^ tubing (catalog #5103K42, McMaster-Carr, Santa Fe Springs, CA, USA) with 1/8″ OD and 0.06″ ID were also evaluated. The different IDs were selected to compare their impact on cartridge performance. Cartridges were fabricated in-house following similar methods to those used for concentrating [^18^F] fluoride, as reported in our previous study [38].

For pinched design cartridges, a 20 cm length of tubing with the desired ID was cut. Small polyethylene (PE) frits (1/8″ thick, 20–micron porosity, Bristol, PA, USA) were punched from a larger disk (catalog #FT20751P, UCT, Inc., Bristol, PA, USA). Punching of frits for the 0.03″, 0.04″, and 0.06″ ID tubing was performed with either 0.03″ (catalog #504529, World Precision Instruments, Sarasota, FL, USA), 0.04″ (catalog #504646, World Precision Instruments), or 0.06″ (catalog #15110–15, Ted Pella Inc., Redding, CA, USA) biopsy punches, respectively. The first frit was inserted and pushed 6 cm into the tubing using a needle-clearing rod obtained from a spinal needle (Quincke Spinal Needle, BD Biosciences, San Jose, CA, USA). The frit was secured by pinching (and plastically deforming) the tubing adjacent to the frit (i.e., prevent the frit from being pushed back out of the tubing when pressure or flow was applied from the opposite end). The cartridge is now ready for filling.

For press-fit design cartridges, two 10 cm lengths of 1/16″ OD 0.03″ ID PTFE tubing were cut. The 1/8″ OD 0.06″ ID tubing was stretched to the point of plastic deformation to slightly reduce its inner diameter (until the 1/16” OD tubing fit snugly inside) and a 4 cm segment was cut. One segment of the 1/16″ OD tubing was inserted 0.5 cm into the larger OD tubing. A frit was inserted into the 1/8″ OD, 0.06″ ID tubing segment until it contacted the end of the 1/16″ OD tubing. The cartridge is now ready for filling.

The desired amount of loose resin was weighed on a balance (Excellence Plus, Mettler Toledo, Columbus, OH, USA) and placed in a 2 mL microcentrifuge tube. A slurry was prepared by adding 0.2 mL of deionized water. Next, the end of the cartridge closest to the frit was connected to a vacuum (−12 psi), and the other end was inserted into the microcentrifuge tube to aspirate the slurry into the tubing. The tube was refilled with 0.2 mL deionized water, and the aspiration process was repeated. This rinsing process was repeated one more time. For pinched design cartridges, after resin loading was complete, a second frit was inserted into the tubing and pushed until it rested against the resin bed, and then the tubing near this second frit was partially pinched to secure both the frits and resin. For press-fit design cartridges, a second frit was inserted into the 1/8″ OD, 0.06″ ID tubing segment and pushed until it rested against the resin. Then, a second piece of 1/16″ OD, 0.03″ ID PTFE tubing was inserted until it rested against the frit. The smaller segments of 1/16″ OD tubing inserted into the larger ID tubing kept the frit in position, eliminating the need for tubing pinching.

All cartridges were preconditioned before use with a 1 mL rinse of deionized (DI) water.

### 4.3. Cartridge Flow Rate Testing

Flow rate measurements were performed to evaluate the impact of cartridge geometry on flow performance and to assess consistency of flow performance across cartridges. In the setup, a sample reservoir (15 mL Falcon conical tube, BD Biosciences, San Jose, CA, USA) was connected to the input of a flow rate sensor (SLI-2000, Sensirion, Westlake Village, CA, USA) via 25 cm of 0.03″ ID, 1/16″ OD tubing, and the cartridge was attached to the output of the sensor. To perform a measurement, 3 mL of DI water was loaded into the sample reservoir, and 20 psi nitrogen pressure was applied, controlled using a manual pressure regulator (ARX21-N01, SMC Corporation, Tokyo, Japan). The resulting flow rate through the cartridge was recorded continuously until the sample reservoir was depleted. The sensor reading was sampled every 74 ms, and the flow rate was estimated by averaging the last 500 samples recorded.

### 4.4. Trapping and Elution Testing

Stock solution of Ga-68 was prepared by diluting a portion of the generator eluate with 0.1 N HCl solution, resulting in activities ranging from 13 MBq to 970 MBq in volumes of 1–10 mL, depending on the experimental need. (For example, for some experiments, a single generator elution was diluted to 10 mL and then divided into 1 mL aliquots to enable multiple experiments to be performed from the same batch of Ga-68.) Three eluents were chosen based on previous reports [28,39,40]: (i) 0.9 N HCl in 90% (*v/v*) EtOH/H_2_O, (ii) 0.05 N HCl in 98% (*v/v*) acetone/H_2_O, and (iii) 0.13 N HCl in 5 M NaCl.

During experiments, the inlet of the cartridge was connected to the bottom of an input vial, to which solutions (Ga-68 stock solution, rinse solution, or elution solution) were manually added. The output of the cartridge was connected to different reservoirs to collect the liquid from different steps in the process. To perform trapping, Ga-68 stock solution was loaded into the input vial and then pushed through the cartridge under positive pressure into the trapping waste vial. Pressurized N_2_ gas was connected to the headspace of the input vial, with pressure controlled via an electronic pressure regulator (ITV0010-2BL, SMC Corporation, Japan) connected through a data acquisition module (DAQ) to a computer running a custom program written in LabView (National Instruments, Austin, TX). Subsequently, a series of elution steps was performed (up to six).

Characterization of trapping and elution efficiency was performed by taking a series of radioactivity measurements using a calibrated dose calibrator (CRC–25 PET, Capintec, Inc., Ramsey, NJ, USA). The following measurements were recorded: the starting activity of Ga-68 (“source”) before trapping (A0_source_), the activity remaining in the source container (e.g., vial or syringe) after trapping (A_source_), the activity in the trapping waste vial after trapping and rinsing (A_waste_), and the collected activity after elution (A_collect_). In some cases, A_collect_ was measured between individual elution steps. The activity trapped on the cartridge, A_cartridge_, was measured indirectly (i.e., calculated as A0_source_ − (A_waste_ + A_source_)) to minimize radiation exposure and because direct measurements of the cartridge were inconsistent, likely due to variations in positioning within the dose calibrator. All radioactivity measurements were decay-corrected to a common time point. 

Trapping efficiency (%) was computed as A_cartridge_/(A0_source_ − A_source_). Elution efficiency (%) was calculated as A_collect_/A_cartridge_. Recovery efficiency (%) was calculated as trapping efficiency × elution efficiency (A_cartridge_/(A0_source_ − A_source_) × (A_collect_/A_cartridge_), describing the total activity recovered after concentration (i.e., factoring in both trapping and elution efficiencies).

### 4.5. Synthesis of [^68^Ga]Ga-PSMA-11

Synthesis of [^68^Ga]Ga-PSMA-11 using concentrated Ga-68 was conducted on a Teflon-coated silicon chip patterned with a circle hydrophilic reaction site, operated on a temperature-controlled heating platform, as previously described [16]. The general synthesis process is depicted in Figure 1. 20 µL of the concentrated Ga-68 in an acetone-based eluent solution was delivered onto a reaction site (preloaded with 10 µL of saline) via micropipette and dried at 60 °C. Additional aliquots of Ga-68 eluate were loaded until the entire batch was used. The result of this process was a concentrated solution of Ga-68 in saline on the reaction site. Next, 10 µL of the PSMA-11 precursor stock solution was added to the same reaction site via micropipette, followed by heating to facilitate complexation. After synthesis, the crude product was collected from the reaction site by adding 20 µL of PBS as a collection solution and then aspirating all liquid via a micropipette and transferring to a 2 mL microcentrifuge tube. This collection step was repeated a total of four times to minimize residual activity on the chip. The crude product was then diluted with 1 mL of DI water for subsequent purification and formulation, during which a 1 µL sample (~16 µCi) was taken for radio-TLC analysis of the crude product. The diluted crude product was loaded onto a C18 cartridge (preconditioned with 3 mL of EtOH followed by 20 mL of DI water) and rinsed with 3 mL of DI water to remove all uncomplexed Ga-68. After drying the cartridge with 20 mL of air via syringe, a 0.22 µm sterile syringe filter was connected to the cartridge, and the final product was eluted from the cartridge with 2 mL of EtOH/DI water (1:1, *v/v*) through the filter into a sterile vial, followed by an additional 14.5 mL of PBS to dilute the EtOH content to acceptable levels.

### 4.6. Analytical EquipmentE and MethodsM

The complexation efficiency of [^68^Ga]Ga-PSMA-11 was evaluated using radio–thin layer chromatography (radio–TLC) based on methods described by Wang et al. [41]. In brief, a 0.5 µL sample was spotted onto a TLC plate (6 cm × 5 cm, cut from 20 cm × 5 cm sheets, silica gel 60 F254, Merck KGaA, Darmstadt, Germany). The plates were developed over a 4 cm distance using a mobile phase consisting of MeOH and 0.1 M ammonium acetate (1:1, *v/v*). After drying, the plates were covered with a glass microscope slide (75 × 50 × 1 mm, Fisher Scientific, Hampton, NH, USA) and imaged by Cerenkov luminescence imaging (CLI) with a 5-min exposure time. The complexation conversion of the sample was determined *via* ROI analysis as previously described [41]. Up to two bands were evident on the TLC: the [^68^Ga]Ga-PSMA-11 product (R_f_ = 0.9–1.0) and unreacted Ga-68 and/or colloids (R_f_ = 0.0). Example radio-TLC images are shown in Figure S1.

The radiochemical yield (RCY) was calculated by comparing the decay-corrected activity of the formulated product to the initial activity of Ga-68 from the generator. The activity yield was determined by the ratio of the activity of the formulated product to the starting activity (not decay-corrected). The radiochemical purity of the final product was assessed using both radio-TLC and radio-HPLC methods. The radio-TLC method was performed as described above. The radio-HPLC method utilized an analytical column (ZORBAX RP Eclipse Plus C18, 100 × 4.6 mm, 3.5 µm, Agilent Technologies, Santa Clara, CA, USA) at a flow rate of 1.2 mL/min under isocratic conditions with a mobile phase of DI water and MeCN (85:15, *v/v*) with 0.1% TFA (*v/v*). The mobile phase was adapted based on the protocol reported by Urbanova et al. [42]. The radio-HPLC system used was a Smartline HPLC system (Knauer, Berlin, Germany) equipped with a degasser (Model 5050), pump (Model 1000), UV detector (206 nm; Eckert & Ziegler, Berlin, Germany), gamma-radiation detector (BFC-4100; Bioscan, Inc., Poway, CA, USA), and counter (BFC-1000; Bioscan, Inc., Poway, CA, USA). The reference standard and co-injection of the reference standard with the final product were analyzed on the same radio-HPLC system to confirm product identity. Example radio-HPLC chromatograms are shown in Figure S2. The apparent specific activity of the formulated [^68^Ga]Ga-PSMA-11 was calculated by dividing the activity of the formulated [^68^Ga]Ga-PSMA-11 by the amount of precursor used.

## 5. Conclusions 

The micro-cartridge-based Ga-68 concentration method developed in this work is rapid, simple, and efficient, providing Ga-68 in microliter volumes with excellent and reliable recovery yields of 98 ± 2% (n = 11; single-generator experiments) and 97 ± 3% (n = 3; dual-generator experiments), with starting Ga-68 activities ranging from 322 to 973 MBq. This method bridges the gap between milliliter-scale radiometal production (up to 4–10 mL) and downstream microscale radiosynthesis using 10s of microliters. In proof-of-concept radiosyntheses of [^68^Ga]Ga-PSMA-11, following concentration with dual-generator-produced Ga-68 (810–837 MBq), radiochemical yield was high (83 ± 11%, n = 3), as was the radiochemical purity (100 ± 0%, n = 3) and apparent specific activity (255–320 GBq/µmol at the end of synthesis). The highly concentrated Ga-68 achieved in this work allows for the use of significantly less precursor (2 µg) than conventional methods while maintaining high performance in radiometal complexation reactions. The possibility to minimize the amount of precursor is particularly beneficial for achieving higher apparent specific activity for applications sensitive to specific activity levels [26,43]. An automated version of this Ga-68 concentration technique is currently in development and could be integrated with an automated microdroplet synthesizer [16,17] for fully automated radiolabeling in a compact microscale radiosynthesizer in the near future.

## Figures and Tables

**Table 1:** Trapping performance of cartridges with different Chromafix PS-H^+^ resin mass (in 0.06″ ID tubing), driving pressure, and fabrication methods. Each data point represents average ± standard deviation for the indicated number of repeats (n).

Cartridge Design	Pinched Design Cartridge (n = 2 Repeats)						Press-Fit Design Cartridge (n = 4)
Resin mass (mg)	5	7	9	12	12	12	12
N_2_ pressure (psi)	12	12	12	12	10	8	6
Trapping Efficiency (%)	76 ± 5	88 ± 2	87 ± 6	94 ± 1	95 ± 1	96 ± 2	100 ± 0
Trapping time (min)	<1	<1	<1	1.3 ± 0.1	1.3 ± 0.0	1.8 ± 0.4	3.5 ± 0.3

**Table 2:** Trap and elution performance for press-fit design tubing cartridges with 12 mg of Chromafix PS-H^+^ resin and 0.05 N HCl in 98% (*v/v*) acetone/H_2_O as eluent. Where applicable, values are presented as the mean ± standard deviation for the indicated number of replicates (n). N.M. = Not measured. E1 = Elution 1. E2 = Elution 2.

	Ga-68 Source				
	Single Generator				Dual Generator
Number of Repeats (n)	2	2	2	11	3
Starting activity (MBq)	311–329	303–315	303–311	322–536	932–973
Trapping efficiency (%)	100 ± 0	100 ± 0	100 ± 0	100 ± 0	100 ± 0
Volume of E1 (µL)	60	60	60	60	60
E1 efficiency (%)	80 ± 4	84 ± 1	83 ± 2	84 ± 7	N.M.
Volume of E2 (µL)	20	30	40	60	60
E1 + E2 efficiency (%)	92 ± 0	94 ± 1	94 ± 3	98 ± 2	97 ± 3

**Table 3:** Comparison of microscale [^68^Ga]Ga-PSMA-11 synthesis conditions and performance to literature reports (microscale and macroscale conditions). Where applicable, values are presented as the mean ± standard deviation for the indicated number of replicates (n). N.R. = Not reported. N/A = Not applicable. R.T. = Room temperature. RCY = Radiochemical yield. RCP = Radiochemical purity. EOS = End of synthesis.

Conditions	This Work	Zhang et al. [24]	Ovdiichuk et al. [27]	Wichmann et al. [29]	Rodnick et al. [30]	Calderoni et al. [31]
Radiosynthesis Platform	Droplet-Based Synthesizer	PDMS Microfluidic Flow Reactor (RAPID Chip)	iMiDEV™ Microfluidic Synthesizer	iPHASE MultiSyn Module (Macroscale)	Scintomics GRP Module (Macroscale)	Cold Kit (Macroscale)
Manual or automated?	Manual	Automated	Automated	Automated	Semi-automated or automated	Manual
Number of repeats (n)	2	3	3	20	>600	N.R.
Starting activity (MBq)	810–837	1110	196–222	1239 ± 156	N.R.	1850
Time for ^68^Ga purification/concentration (min)	~4.5	~12	2–5	N/A	~8	N/A
Precursor amount (μg)	2	1–2	10	10	10	25
Precursor amount (nmol)	1.98 *^a^*	0.99–1.98 *^a^*	9.88 *^a^*	9.88	9.88 *^a^*	24.7 *^a^*
Precursor concentration (μM)	~132 *^a^*	2.5–5.0 *^a^*	19.8 *^a^*	1.9	3.1 *^a^*	<4.9 *^a^*
Reaction volume (μL)	~15	400	500	5100	3200	>5000
Reaction temperature (°C)	95	95	95	95	125 (setting)	R.T.
Reaction time (min)	1	1	1	5	10	5
Complexation efficiency (%) *^b^*	100 ± 0	99–100	98.1 ± 0.7	96.6 ± 0.6	N.R.	>98
Purification and reformulation?	Yes	No	Yes	Yes	Yes	No
RCY (%)	83 ± 11	70	46.5 ± 2.6	76 ± 3	N.R.	99.9
Activity yield (%)	73 ± 10	62 *^c^*	38 ± 2 *^c^*	64 ± 3 *^c^*	N.R.	90 *^c^*
Activity yield (GBq)	0.51–0.64	0.69 *^c^*	0.08–0.09 *^c^*	0.69–0.89 *^c^*	0.52–1.78	1.67 *^c^*
RCP (%)	100 ± 0 *^d^^e^*	>99 *^d^*	98.5–100 *^d^*	99.9 ± 0.2 *^d^*	>99 *^d^^e^*	>98 *^d^*
Apparent specific activity at EOS (MBq/μg)	255–320	>740 *^f^*	8–9 *^c^*	69–89 *^cg^*	52–178 *^c^*	67 *^c^*
Overall radiosynthesis time (min)	12 ± 0	12–15	~19	17	35	10

*^a^* The molecular weight of the PSMA-11 precursor is often not reported because it is supplied as a trifluoroacetate salt, with varying trifluoroacetate content between batches. For purposes of making comparisons of precursor concentration in μM units, we converted values using the molecular weight reported by Wichmann et al. [29] (1012.1 g/mol). Thus, the calculated mole amounts should be considered as estimates only. *^b^* The complexation efficiency was determined by radio-TLC. *^c^* The value was calculated based on details in the literature report. *^d^* The RCP was determined by radio-HPLC. *^e^* The RCP was determined by radio-TLC. *^f^* For this measurement, 1 μg of precursor was used. *^g^* This value is calculated based on the amount of precursor to be consistent with other reports. The authors reported a different value in the paper (>782 ± 99 MBq/μg), which was calculated using a calibration curve of reference standard on an analytical HPLC.

**Table 4:** Comparison of the conditions and performance of the developed Ga-68 concentration methods with previously reported microfluidic-based concentration approaches. Where applicable, values are presented as the mean ± standard deviation for the indicated number of replicates (n). N.R. = Not reported.

Method	This Work		Zhang et al.	Mallapura et al.
	Pinched Design Micro-Cartridge (0.04″ ID)	Press-Fit Design Micro-Cartridge (0.06″ ID)	Resin Packed Into Microchannel	Resin Packed Into Microchamber
Manual or automated?	Semi-automated	Semi-automated	Automated	Automated
Number of generators used	1	1–2	1	1
Volume of ^68^Ga (mL)	6	6.0–6.4	4	5
Starting activity (MBq)	760–970	322–973	1110	1100–1200
Resin type (amount)	SCX (9 mg)	SCX (12 mg)	SCX (8 mg)/ SAX (10 mg)	SCX (50 µL)
Same direction or reverse trap/release	Same direction	Same direction	Same direction	Reverse direction
Elution solution	0.05 N HCl in 98% (*v/v*) acetone/H_2_O		DI water	0.15 M HCl in 5 M NaCl
Recovered elution volume (µL)	60	80	200	200
Number of repeats (n)	2	11 *^a^* 3 *^b^*	5	4
Trapping efficiency (%)	100 ± 0	100 ± 0	96.5 ± 1.6 (SCX) 89.5 ± 3.0 (SAX) *^c^*	>98.5
Recovery efficiency (%)	93 ± 0	98 ± 2 *^a^* 97 ± 3 *^b^*	73.7 ± 3.8	91 ± 6 *^d^*
Total process time (min)	~9	~4.5	~12	N.R.

*^a^* Using output of one generator. *^b^* Using output of two generators. *^c^* The trapping efficiency on the SAX cartridge was based on the amount of activity eluted out from the SCX cartridge. *^d^* The recovery efficiency is slightly overestimated as it was calculated based on the trapped activity rather than the starting activity before trapping.

## Data Availability

Data are contained within the article and Supplementary Materials.

## Supplementary Materials

The following are available online at https://www.mdpi.com/article/10.3390/molecules29194572/s1. Table S1: Flow rates of cartridges fabricated with different resin, resin mass, and tubing ID; Table S2: Summary of trapping and elution performance for various-sized cartridges packed with Oasis MCX resin; Table S3: Performance of trapping and elution with different cartridges (resin type and resin mass) packed in 0.04″ ID tubing and different types of elution solutions; Table S4: Trap and elution performance for pinched design tubing cartridges with 9 mg of Chromafix PS-H^+^ resin packed in 0.04″ ID tubing and 0.05 N HCl in 98% (*v/v*) acetone/H_2_O as an eluent solution; Mock radiosynthesis; Table S5: Performance of microscale [^68^Ga]Ga-PSMA-11 synthesis (n = 3 replicates) with concentrated Ga-68 produced by dual generators; Figure S1: Example of Cerenkov luminescence image of radio-TLC; Figure S2: Example radio-HPLC chromatograms.
