# Supplementary material for: Rapid Concentration of Ga-68 and Proof-of-Concept Microscale Labeling of [68Ga]Ga-PSMA-11 in a Droplet Reactor
Source: Molecules. 2024 Sep 26;29(19):4572. doi: 10.3390/molecules29194572 (PMC11477945; doi:10.3390/molecules29194572)
Supplement: Supplementary file 1 [file molecules-29-04572-s001.zip › molecules-3208209-supplementary.pdf]

molecules

Supplementary Information

Rapid Concentration of Ga-68 and Proof-of-Concept Microscale Labeling of [^{68}Ga]Ga-PSMA-11 in a Droplet Reactor

1. Preliminary flow rate study

Table S1. Flow rates of cartridges fabricated with different resin, resin mass, and tubing ID. Each data point represents average ± standard of n = 500 flow rate measurements measured at 74 ms intervals.

Oasis MCX Resin						
Resin mass (mg)	3	3	5	5	7	9
Tubing ID (inch)	0.03	0.04	0.03	0.04	0.04	0.04
Flow rate (μL/min)	1020 ± 10	3060 ± 20	630 ± 0	2000 ± 10	1580 ± 20	820 ± 10
**Chromafix PS-H^+ Resin**						
Resin mass (mg)	7	9	9	11	15	
Tubing ID (inch)	0.04	0.04	0.06	0.06	0.06	
Flow rate (μL/min)	2630 ± 30	1470 ± 20	4330 ± 40	3800 ± 20	3080 ± 20	

2. Preliminary study comparing cartridge performance for different geometries

Table S2. Summary of trapping and elution performance for various sized cartridges packed with different amounts of Oasis MCX resin. Each condition was measured once (n = 1), and solutions were manually pushed through the cartridge with a syringe (at ~1 mL/min).

Tubing ID (inch)	0.03	0.04	0.04	0.04	0.06	0.06	0.06
Resin mass (mg)	3	3	5	7	9	11	15
Trapping Efficiency (%)	83	81	90.	94	99	98	99
Elution Efficiency (%)	94	71	63	70	29	35	20

3. Performance of trapping and elution with narrow cartridges (packed in 0.04″ ID tubing)

Table S3. Performance of trapping and elution with different cartridges (resin type and resin mass) packed in 0.04″ ID tubing and different types of elution solutions. Each data point represents average ± standard deviation of n = 2 repeats. E1, E2, E3, etc. refer to the first, second, third, etc. elution steps, each performed with 10 μL volume of eluent solution.

**Eluent type**	**0.05N HCl in 98% (v/v) acetone/H_2O**				**0.13N HCl in 5M NaCl**		**0.9N HCl in 90% (v/v) EtOH/ H_2O**	
**Resin type**	**Chromafix PS-H^+**	**Oasis MCX**	**Chromafix PS-H^+**	**Oasis MCX**	**Chromafix PS-H^+**	**Oasis MCX**	**Chromafix PS-H^+**	**Oasis MCX**
Resin mass (mg)	7	7	9	9	9	9	9	9
Trapping efficiency (%)	97 ± 1	99 ± 0	99 ± 0	99 ± 0	99 ± 1	99 ± 0	99 ± 0	99 ± 0
E1+E2 efficiency (%)	5 ± 6	6 ± 2	12 ± 20	1 ± 0	28 ± 27	10 ± 3	2 ± 1	1 ± 1
E3+E4 efficiency (%)	69 ± 10	53 ± 5	55 ± 3	24 ± 2	40 ± 0	3 ± 3	2 ± 3	0 ± 0
E5+E6 efficiency (%)	22 ± 8	31 ± 7	28 ± 15	42 ± 1	19 ± 10	4 ± 5	6 ± 7	0 ± 0
E1 to E4 efficiency (%)	74 ± 8	59 ± 7	68 ± 14	25 ± 2	68 ± 30	12 ± 6	4 ± 2	1 ± 1
E1 to E6 efficiency (%)	96 ± 0	90 ± 0	96 ± 1	67 ± 1	87 ± 10	16 ± 10	10 ± 9	1 ± 1

Table S4. Trap and elution performance for pinched design tubing cartridges with 9 mg of Chromafix PS-H^+ resin packed in 0.04″ ID tubing, and 0.05N HCl in 98% (v/v) acetone/H_2O as an eluent solution. Each data point represents average ± standard deviation of the indicated number of repeats (n). E1, E2, E3, etc. refer to the first, second, third, etc. elution steps, each performed with 10 μL volume of eluent solution.

**Starting Activity (MBq)**	**37–74**	**440**	**760–970**
Number of repeats (n)	2	1	2
Volume of Ga-68 (mL)	10	10	6
Trapping efficiency (%)	99 ± 0	99	100 ± 0
E1+E2 efficiency (%)	9 ± 1	5	2 ± 1
E3+E4 efficiency (%)	71 ± 4	75	72 ± 20
E5+E6 efficiency (%)	16 ± 5	12	20 ± 20
E1 to E4 efficiency (%)	80. ± 5	81	74 ± 20
E1 to E6 efficiency (%)	96 ± 0	93	93 ± 0

4. Mock radiosynthesis

To determine some characteristics of the microscale radiosynthesis process, we initially performed a "cold run", including a mock transfer of concentrated Ga-68 (using just the eluent solution) to the chip, and mock droplet radiosynthesis (by adding precursor solution).

First, 10 μL of saline was loaded on the chip reactor, followed by 4 portions (20 μL each) of acetone-based eluent, each heated at 60°C after loading to evaporate the acetone. After the final step, the reactor was cooled, and a micropipettor (0.1–10 μL range) was used to manually measure the residual volume by adjusting the pipettor's volume setting to match the residue on the chip. This process was repeated three times to obtain an average residual volume.

After the mock evaporation of the acetone-based eluent on the chip reactor, 10 μL of precursor stock solution was added. To measure the pH of the reaction mixture following radiosynthesis, the mixture was heated at 95°C for 1 min, then cooled down to room temperature before taking a 1 μL sample for pH measurement with a pH test strip. This process was repeated three times.

5. Performance of microscale [^{68}Ga]Ga-PSMA-11 synthesis

Table S5. Performance of microscale [^{68}Ga]Ga-PSMA-11 synthesis (n = 3 replicates) with concentrated Ga-68 produced by dual generators.

**Batch**	**1**	**2**	**3**	**Average ± standard deviation**
**Concentration Step**				
Starting activity (A_0, MBq)	836	810	837	828 ± 15
Trapping efficiency (%, based on A_0)	99.6	99.2	99.7	100 ± 0
Elution efficiency (%,based on A_0)	98.0	94.2	99.0	97 ± 3
Recovery efficiency (%, based on A_0)	97.6	93.4	98.3	96 ± 3
Concentrated activity (A_{conc}, MBq)	792	699	744	745 ± 47
Duration (min)	4.5	4.0	4.5	4.3 ± 0.3
**Radiolabeling Step**				
Concentrated activity transferred to chip reactor (A_{0_chip}, %)	73.9	99.2	92.2	88 ± 13
Collected crude activity after reaction (% based on A_{0_chip})	98.0	98.3	99.1	99 ± 1
Collected crude activity after reaction (A_{crude}, MBq)	544	681	677	634 ± 78
Duration (min)	4	4	4	4 ± 0
**Purification and reformulation Step**				
Residual activity in collection vial after purification (%, based on A_{crude})	0	1	0	0 ± 1
Waste from C18 trapping and rinse (%, based on A_{crude})	1	0	0	0 ± 1
Elution efficiency from C18 (%, based on A_{crude})	98	99	99	99 ± 1
Formulated product (MBq)	511	648	643	601 ± 78
Residual activity in C18 and filter (%, based on A_{crude})	1	1	1	1 ± 0
Duration (min)	3.5	3.5	3.5	3.5 ± 0.0
**Overall radiosynthesis time (min)**	12	11.5	12	12 ± 0

6. Example radio-TLC images

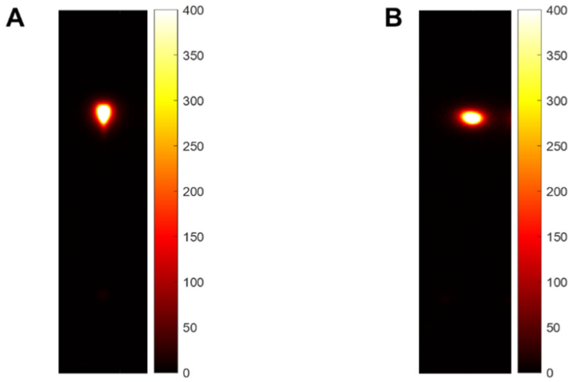

Figure S1. Example of Cerenkov luminescence image of radio-TLC of (A) crude product after ^{68}Ga complexation and (B) purified and formulated [^{68}Ga]Ga-PSMA-11.

7. Radio-HPLC analysis of [^{68}Ga]Ga-PSMA-11

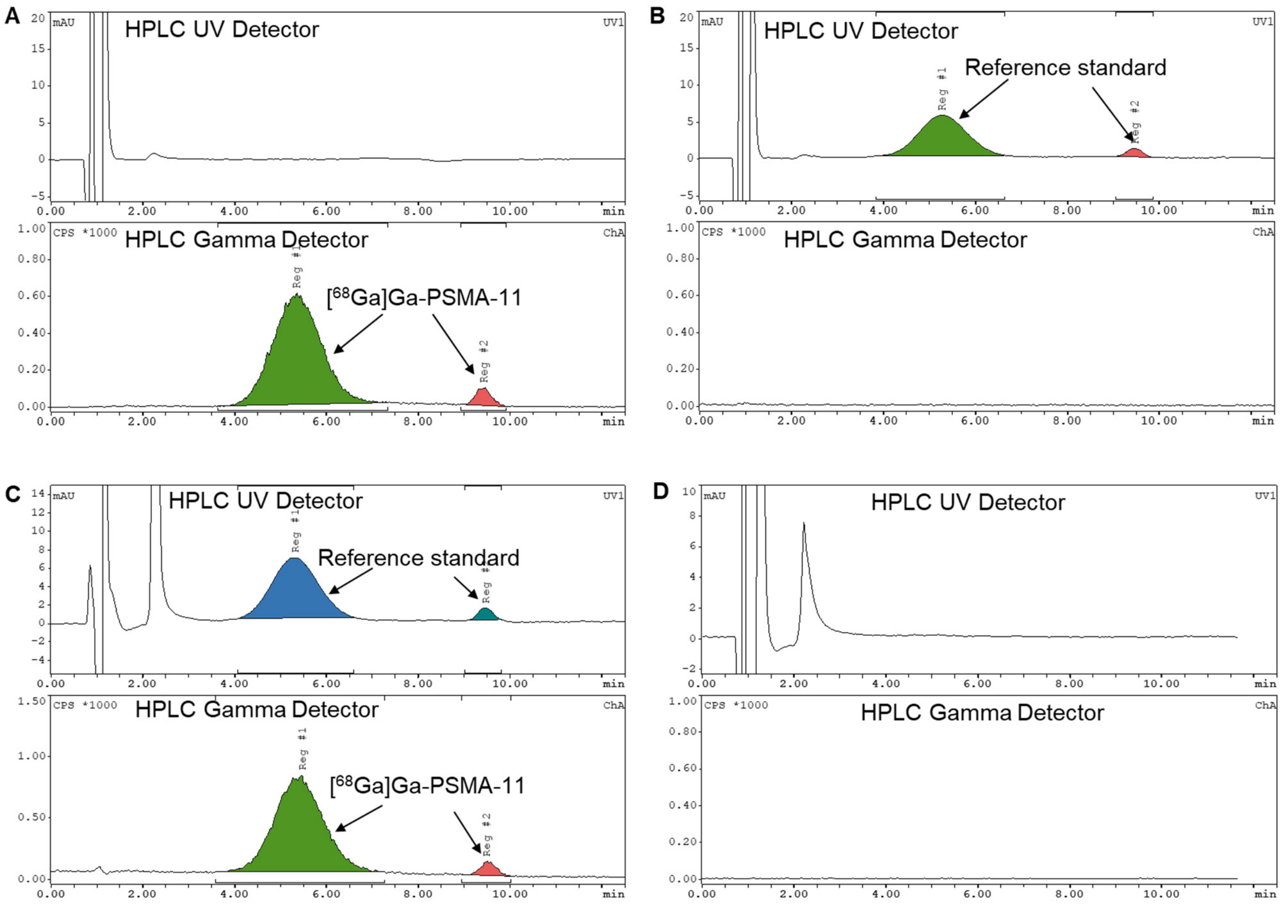

Figure S2. Example radio-HPLC chromatograms. (A) Purified [^{68}Ga]Ga-PSMA-11 from microscale radiosynthesis. (The two diastereomers were separated using an isocratic analytical method, similar to a previous report by Urbanova et al. [1]). (B) Reference standard. (C) Co-injection of purified [^{68}Ga]Ga-PSMA-11 and reference standard. (D) Blank injection of formulation buffer (i.e., PBS:EtOH = 94:6 (v/v)).

References

1. Urbanová, K.; Seifert, D.; Vinšová, H.; Vlk, M.; Lebeda, O. Simple New Method for Labelling of PSMA-11 with 68Ga in NaHCO3. *Applied Radiation and Isotopes* **2021**, *172*, 109692, doi:10.1016/j.apradiso.2021.109692.
